# Increased Vascularization in the Vulnerable Upstream Regions of Both Early and Advanced Human Carotid Atherosclerosis

**DOI:** 10.1371/journal.pone.0166918

**Published:** 2016-12-14

**Authors:** Ola Hjelmgren, Karl Gellerman, Josefin Kjelldahl, Per Lindahl, Göran M. L. Bergström

**Affiliations:** 1 The Wallenberg Laboratory, Department of Molecular and Clinical Medicine, Institute of Medicine, Sahlgrenska Academy at the University of Gothenburg, Gothenburg, Sweden; 2 Department of Clinical Physiology, Sahlgrenska University Hospital, Gothenburg, Sweden; Universitatsklinikum Freiburg, GERMANY

## Abstract

**Background:**

Vascularization of atherosclerotic plaques has been linked to plaque vulnerability. The aim of this study was to test if the vascularization was increased in upstream regions of early atherosclerotic carotid plaques and also to test if the same pattern of vascularization was seen in complicated, symptomatic plaques.

**Methods:**

We enrolled 45 subjects with early atherosclerotic lesions for contrast enhanced ultrasound and evaluated the percentage of plaque area in a longitudinal ultrasound section which contained contrast agent. Contrast-agent uptake was evaluated in both the upstream and downstream regions of the plaque. We also collected carotid endarterectomy specimens from 56 subjects and upstream and downstream regions were localized using magnetic resonance angiography and analyzed using histopathology and immunohistochemistry.

**Results:**

Vascularization was increased in the upstream regions of early carotid plaques compared with downstream regions (30% vs. 23%, p = 0.033). Vascularization was also increased in the upstream regions of advanced atherosclerotic lesions compared with downstream regions (4.6 vs. 1.4 vessels/mm2, p = 0.001) and was associated with intra-plaque hemorrhage and inflammation.

**Conclusions:**

Vascularization is increased in the upstream regions of both early and advanced plaques and is in advanced lesions mainly driven by inflammation.

## Introduction

In the atherosclerotic plaque, the regions upstream and downstream of the maximum stenosis will be exposed to different hemodynamic forces. High wall shear stress characterizes the upstream region while the downstream region is associated with low wall shear stress and turbulent blood flow[1]. Different types of shear stress might induce different gene expression patterns in endothelial cells[2], possibly leading to differences in plaque phenotype. In fact, our group and others have shown that the upstream region of the advanced carotid plaque exhibits a more vulnerable phenotype and is often the site of plaque rupture[3–7]. Vascularization of the plaque has been linked to plaque vulnerability and progression, possibly by providing an entry point for inflammatory cells, causing intra-plaque hemorrhage (IPH), inflammation and subsequent destabilization[8–10]. Two studies reported that plaque vascularization was increased in the upstream region[5, 7], but only the shoulder regions of the plaques were studied and the exact relation of the tissue sections to the point maximum stenosis were not reported. Furthermore, those studies relied on *ex vivo* assessment of endarterectomy specimens from patients with late stage, advanced atherosclerotic disease. Upstream and downstream vascularization has not yet been measured *in vivo* and whether there are differences along the longitudinal axis early in plaque development is not known.

Plaque vascularization is thought to be driven by hypoxia (mediating its effects through Hypoxia-Inducible Factor 1 alpha, HIF1-α), but also by inflammation itself[8]. During hypoxia, HIF-1α up-regulates the expression of the key angiogenic molecule Vascular Endothelial Growth Factor (VEGF) and its receptor (VEGFR2), but VEGF and VEGFR2 may also be induced in a hypoxia-independent manner by inflammation[11]. Adequate vessel formation is further dependent on the complex interactions between VEGF, its receptors and the Notch signaling pathway[12, 13]. However, the Notch pathway is also present in inflammatory processes[14], and may thus play a part in the inflammatory component of atherosclerosis.

The aim of this study was to investigate the distribution of vessels between upstream and downstream regions of early and late stage human carotid plaques *in vivo* and *ex vivo* using contrast enhanced ultrasound (CEUS) and histology/immunohistochemistry respectively. The upstream and downstream regions were carefully identified in relation the point of maximum stenosis using in vivo ultrasound or Magnetic Resonance Angiography (MRA). We also aimed to determine whether hypoxia or inflammation is the main driving force for vascularization in late stage atherosclerotic plaques and to describe the expression of key angiogenic proteins in the upstream and downstream regions.

## Materials and Methods

### Study population

The selection of early stage plaque patients for CEUS and late stage plaque patients, for immunohistochemistry have been described in detailed elsewhere[6, 15]. For CEUS examination, subjects, less than 80 years of age, were recruited from the Western region Initiative to Gather Information on Atherosclerosis (WINGA) database as previously described[15]. This database includes patients at Sahlgrenska university hospital undergoing ultrasound examination for suspected cerebrovascular disease. We also invited volunteers aged 68–73 years (identified through official registers) to a screening program for carotid artery atherosclerosis. In this study, subjects with a plaque localized in the common or internal carotid artery with a minimum height of 2,5 mm was selected. Patients with more than one plaque were excluded.

The study sample for the immunohistochemistry consisted of symptomatic carotid atherosclerotic plaques obtained from the Gothenburg Atheroma Study Group (GASG) bio bank of patients who underwent carotid endarterectomy (CEA) at the Sahlgrenska University Hospital (Gothenburg, Sweden) between October 2003 and April 2008. Criteria qualifying for surgery were minor ischemic stroke, transient ischemic attack (TIA) or amaurosis fugax (AFX), and a high-grade carotid stenosis (≥ 70% according to the ECST[16]). From this population, we consecutively included patients who had undergone MRA as part of the pre-operative investigation and who were available for blood sampling. The local clinical work up for these patients is very fast[17] and it was therefore not feasible to perform a CEUS examination before CEA.

All subjects were asked to complete a questionnaire to provide clinical and lifestyle data and venous blood samples were drawn as previously described[18]. This study conforms to the Declaration of Helsinki and was approved by the regional ethics board in Gothenburg. All subjects gave written informed consent.

### Ultrasound protocol

The early stage plaque subjects were imaged using a Siemens S2000, update VA16D, ultrasound platform (Siemens, Mountain View, California, USA) equipped with a 9L4 probe. We used 9MHz for B-mode ultrasound imaging and 4 MHz for cadence imaging. The carotids were scanned and the largest plaque was selected for contrast enhanced ultrasound imaging using a standardized protocol[15]. In short, a longitudinal image plane was selected, cutting through the center of the vessel and through the maximum thickness of the plaque. Using cadence contrast imaging (CPS) for Siemens S2000 we recorded a 150s loop after injection of 1.6ml of contrast-agent (Sonovue, Bracco imaging, Milan, Italy). We used standardized image settings, low mechanical index (0.06) and low gain.

### CEUS-Image acquisition and analysis

**Fig 1:**
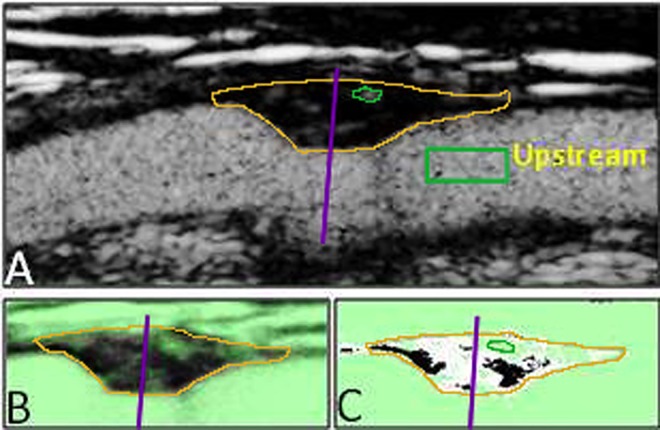
CEUS image of the carotid artery (A) with a plaque in the near wall. The plaque border is marked with orange and the upstream/downstream divider as a purple line (lines have been enhanced for clarification). B: CQP image with the mean pixel intensity. Automatically excluded areas marked green. C: CQP image with pixels with mean intensity above threshold level marked white.

All CEUS images were handled and analyzed using the Contrast Quantification Program (CQP)[15]. We used Sonovue, an ultrasound contrast agent made up of micro bubbles which are strictly localized to the vascular compartment. When contrast agent is visualized inside the boundaries of a plaque it is therefore a sign of plaque vascularization. In brief, the CQP calculates the percentage of plaque area in a longitudinal section which contains contrast agent and this is used as an index of vascularization (CQP-value, %). The upstream and downstream part of the plaque image was then manually identified using a divider line. The separation in upstream and downstream regions was made blinded for information on contrast agent uptake using non-contrast containing images. To measure the inter observer variability in the positioning of the divider, two observers (KG and OH) who were blinded for each other placed the divider through the maximum stenosis of 23 plaques. The area downstream of the divider was used to measure the difference between the observers, revealing a strong correlation (Spearman’s rho = 0.85, p<0.001) and no systematic difference between them (p = 0.12). One experienced sonographer evaluated the contrast enhanced ultrasound images (OH). The CQP software then calculated the CQP-value in both the upstream and downstream part of the plaque respectively (Fig 1).

### Preoperative imaging

In the group with advanced plaque disease, MRA examinations were performed in a Philips Gyroscan Intera 1.5, Release 9 unit with a head and neck coil (Philips Medical Systems, Best, The Netherlands). In the in vivo MRA images, the distance from the flow divider to the site of maximum stenosis was measured.

### Tissue processing and selection of upstream and downstream section

After surgical removal, endarterectomies were immediately fixed in formalin. From along the whole longitudinal axis of the plaque, 3-mm thick tissue blocks were prepared and for each block the distance from the flow divider in the bifurcation was measured. To determine the distance from the maximum stenosis for each tissue block the distance from the bifurcation was related to the MRA measurement. As we have reported previously, there is a good agreement between the point of maximum stenosis evaluated by MRA and the narrowest lumen found by histopathological assessment[6]. From each tissue block, serial transverse 4-μm sections were prepared for histopathological and immunohistochemical analysis. One upstream section (US) and one downstream section (DS) was assigned for each plaque so that their respective distance to the point of maximum stenosis should be equal. Only sections located within 1.5 to 7.5 mm from the point of maximum stenosis were considered. The US/DS pair of sections was selected to generate the least possible difference in distance to the point of maximum stenosis between the US and DS sections. The difference in distance to the point of maximum stenosis did not exceed 1.5 mm.

### Histopathological assessment

Histological staining and histopathological classification, including the American Heart Association (AHA) classification[19], were performed as described previously[6]. All histological classification was done by one author (JK) and blinded to all other data. To test the reproducibility of the classification, 89 sections from 30 carotid plaques separate from this study were analyzed. The same observer (JK), classified the same image twice with an interval of 2 weeks, showing a good reproducibility (spearman’s rho = 0.82, p = 0.001). The second reading was blinded to the first reading.

In addition to AHA classification, classification regarding presence of the different components of the AHA classification was also performed (thin fibrous cap, intra plaque hemorrhage, plaque rupture and/or intraluminal thrombosis). The histopathological definitions used were adopted from Lovett et al.[20] Immunohistochemical staining was done with mouse monoclonal antibodies against CD34 as endothelial cell marker (DAKO, Glostrup, Denmark; 1:25 dilution), HIF1α (Novus Biologicals, Littleton, CA; 1:1000 dilution), VEGF (Serotec, Oxford, UK; 1:1000 dilution), VEGFR-2 (Serotec; 1:100 dilution), Dll4 (Serotec; 1:200 dilution), Notch1 (Epitomics, Burlingame, CA; 1:100 dilution). Immune complexes were visualized using the MACH3 Mouse-Probe Alk Phos Polymer Kit (BioCare Medical, Concord, CA) together with the Vulcan Fast Red Chromogen kit (BioCare Medical). Primary antibody incubation was performed in room temperature for 1 hour. Immunohistochemical staining of macrophages (anti-CD68) was done as described previously[6]. Stained sections were digitalized using a Zeiss Mirax Scanner (Zeiss, Jena, Germany). Digital images were analyzed using the BioPix software (BioPix AB, Gothenburg, Sweden).

Extent of immunohistochemical staining is expressed as percentage stained area of the total section area (lipid core and lumen were excluded). CD34^+^ vessel density was analyzed at high magnification (x40) and was assessed on the entire tissue sections and expressed as number of vessels per total section area excluding the lipid core.

### Statistical analysis

IBM SPSS Statistics 20.0 for Windows (IBM, Armonk, US-NY) was used for analyses. Results are given as means (SD) for parametric or medians (quartiles) for non-parametric variables. Categorical variables are presented as counts (%). Upstream and downstream sections where treated as related samples when compared. Parametric and non-parametric methods were used as appropriate. Correlations were tested using Spearman’s rho. Two-sided p-values <0.05 were considered significant.

## Results

### Study population and plaque histology

Fifty-two patients were recruited to the CEUS examination. Seven patients were later excluded because they had more than one visible plaque (Table 1).

**Table 1:** Patient characteristics.

	n	CEA patients	n	CEUS patients
Age, years	56	68.6 (8.8)	45	68.2 (6.6)
Male sex,	56	43 (77%)	45	30 (67%)
Ipsilateral stroke or TIA		56 (100%)	45	10 (22%)
Days between event and surgery	56	78 (46, 104)		N/A
Type of event	56			N/A
AFX		7 (13%)		
TIA		11 (20%)		
Stroke		38 (68%)		
Diabetes	56	14 (25%)	45	4 (9%)
Hypertension	56	39 (70%)	45	27 (60%)
Smoking*	56	11 (20%)	45	9 (20%)
Previous CHD†	53	6 (11%)	45	3 (7%)‡
Cholesterol, mmol/l	55	4.9 (1.2)	45	5.4 (1.2)
LDL, mmol/l	55	2.7 (1.1)	45	3.2 (1.1)
HDL, mmol/l	55	1.3 (0.4)	45	1.8 (0.5)
Triglycerides, mmol/l	55	2.1 (1.0)		N/A
Statin treatment	56	45 (80%)	42	13 (29%)
ACE or ARB inhibitors	56	22 (39%)		N/A
Anti-platelet medication	56	51 (91%)	42	13 (29%)
Warfarin	56	4 (7%)	39	2 (4%)
Diabetes medication	56	11 (20%)		4 (9%)

*Within same year as inclusion.

†Myocardial infarction a/o angina pectoris.

‡Myocardial infarction only.

During the study period, plaques from 265 patients were collected and 56 of these fulfilled the inclusion criteria (blood sampling and MRA). The included patients were more likely to have stroke as qualifying event (68 vs. 42%, p = 0.001), be on lipid lowering medication (80 vs. 67%, p = 0.048) and had a longer time between their clinical event and surgery (Median 78 vs. 32 days, p<0.001). Characteristics of the included patients are presented in Table 1.

As presented in Table 2, sections from the upstream region had more vulnerable plaque characteristics; upstream regions were more likely to be AHA class VI while downstream regions were significantly more often AHA III.

**Table 2:** Histological plaque features and immunostaining in upstream and downstream regions.

	n	Upstream	n	Downstream	p
AHA class	56		52		
III		2 (4%)		12 (23%)	0.006
IV		19 (34%)		14 (27%)	0.33
V		11 (20%)		18 (35%)	0.077
VI		24 (43%)		8 (15%)	0.001
Intra plaque hemorrhage	56	38 (68%)	52	16 (31%)	<0.001
Thrombosis	56	19 (34%)	52	7 (14%)	0.006
Thin fibrous cap	56	43 (77%)	52	22 (42%)	<0.001
Rupture	56	24 (43%)	52	8 (15%)	0.001
Number of vessels, n/mm^2^ (quartiles)	54	4.6 (1.6–10.2)	55	1.4 (0.3–5.7)	0.001
CD68 stained area, %	56	6.3 (1.3–17.1)	56	2.3 (0.4–11.4)	0.007
HIF1-α stained area, %	56	6.2 (4.0–9.0)	56	6.9 (5.4–9.9)	0.009
VEGF stained area, %	56	1.5 (0.4–3.9)	56	0.4 (0.1–1.1)	<0.001
VEGFR2 stained area, %	56	1.1 (0.4–2.1)	55	0.5 (0.2–1.0)	<0.001
Dll4 stained area, %	56	1.0 (0.5–1.7)	56	0.6 (0.2–1.5)	0.031
Notch stained area, %	56	1.4 (0.6–2.2)	56	0.9 (0.4–1.5)	0.026

### Increased number of vessels in the upstream region of both early and late stage plaques

**Fig 2:**
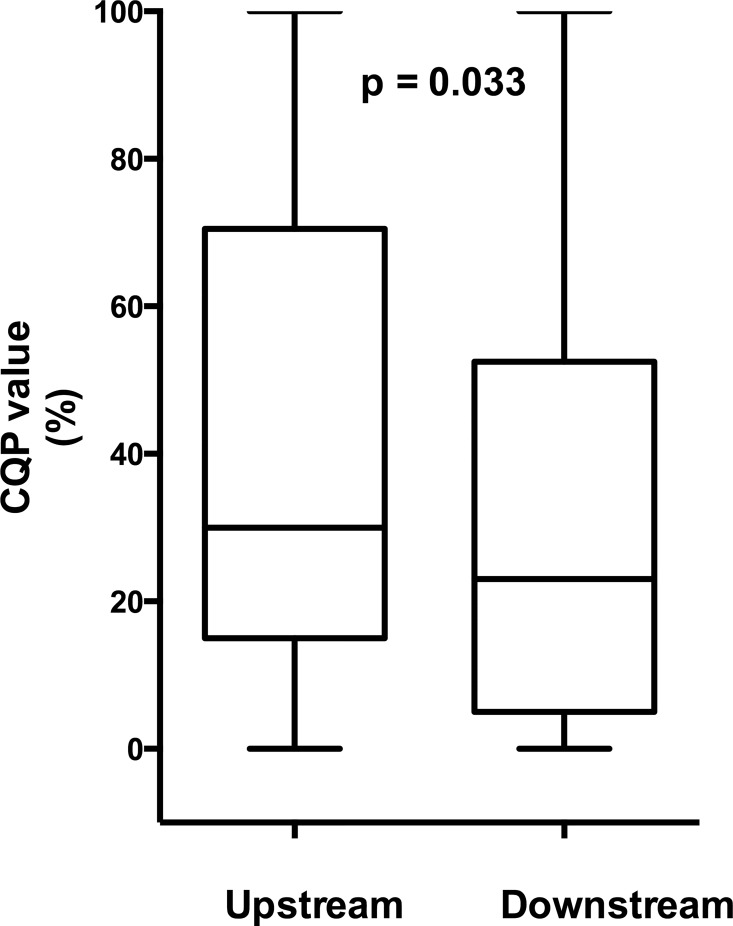
Boxplot of vascularization, measured as CQP values in upstream and downstream parts of early stage plaques.

In the early stage plaque group, the median CQP value in the plaques was 30% (11–54). As reported earlier, plaques in the far wall and plaques from patients with previous ipsilateral stroke or TIA had higher CQP-values[15]. In the upstream region, the median CQP-value was 30% (15–69), which was significantly higher, p = 0.033, compared with the downstream region, 23% (6–52), (Fig 2). In patients on lipid lowering medication (n = 13) there was a trend towards relatively higher CQP values downstream compared with patients not on lipid lowering medication (n = 29). This trend was not seen in the upstream part. The median difference between upstream and downstream sections was -6% (-16% - 2%) in patients on lipid lowering medication and 4% (0% - 23%) not on medication (p = 0.037). However, the low number of observations makes this finding uncertain. No other associations were found between CQP-values or its distribution between upstream and downstream regions and age, sex, diabetes, far-wall plaques or previous ipsilateral stroke or TIA (data not shown).

**Fig 3:**
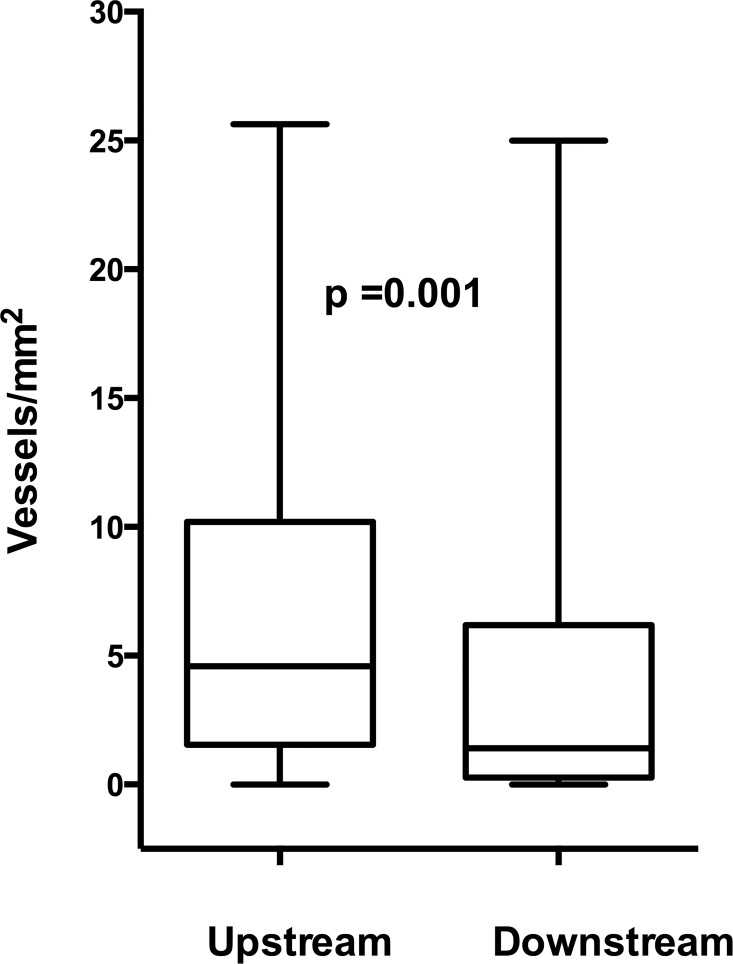
Boxplot of the number of CD34-stained vessels/mm^2^ in upstream and downstream parts of advanced plaques.

In the group with advanced plaque disease, the number of vessels in the endarterectomy specimens was increased in the upstream region compared with the downstream region, 4.6 (1.6–10.2) vs. 1.4 (0.3–5.7) vessels/mm^2^, p = 0.001 (Fig 3). Sections with IPH had increased number of vessels in both upstream, 5.2 (3.5–10.5) vs. 1.7 (0.0–5.8) vessels/mm^2^, p = 0.020 and downstream sections, 3.4 (1.4–5.6) vs. 0.8 (0.0–5.7), p = 0.036 compared with sections with no IPH. No associations were found between the number of vessels or vessel distribution between upstream and downstream regions and time since clinical event, lipid lowering medication, diabetes, age, sex or type of clinical event (data not shown).

### Increased inflammation but decreased Hif1-α staining in the upstream region

As presented in Table 2, the CD68-stained area were larger in the upstream regions, median area 6.3% (1.3–17.1)) compared with downstream regions, 2.3% (0.4–11.4), p = 0.007. Hif1-α staining showed the opposite pattern, 6.2% (4.0–9.0) vs. 6.9% (5.4–9.9), p = 0.009. There was a positive correlation between vessel density and CD68 staining in both the upstream and downstream regions, ρ = 0.52, p<0.001 and ρ = 0.48, p<0.001 respectively, while no correlation was found between vessel count and Hif1-α in upstream or downstream regions, ρ = -0.02, p = 0.90 and ρ = -0.04, p = 0.76 respectively.

**Fig 4:**
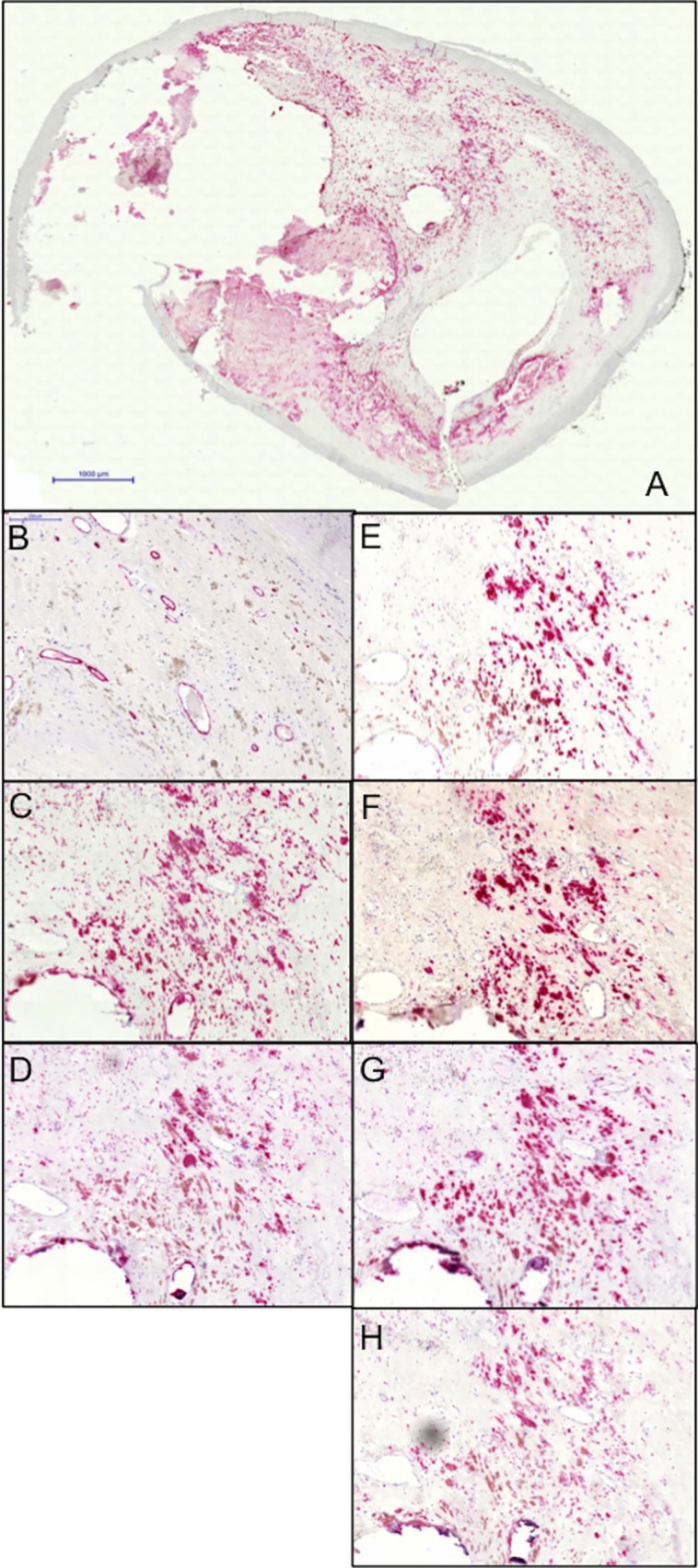
Example of immunostaining of different antibodies. A: Overview. B: CD34 stained vessels. C: CD68 positive macrophages. D: HIF1α. E: VEGFR2. F: VEGF. G: NOTCH1. H: Dll4. Black bars represent 1000 μm (A) and 200 μm (B-H).

VEGF, VEGFR2, Notch1 and Dll4 were all significantly increased in the upstream region (Table 2). VEGF, VEGFR2, Notch1 and Dll4 expression was mainly localized to areas with intense CD68 stained cells, while the intensity of these markers were less evident in CD34+ endothelial cells (Fig 4).

### Differences in protein expression across AHA classes

**Fig 5:**
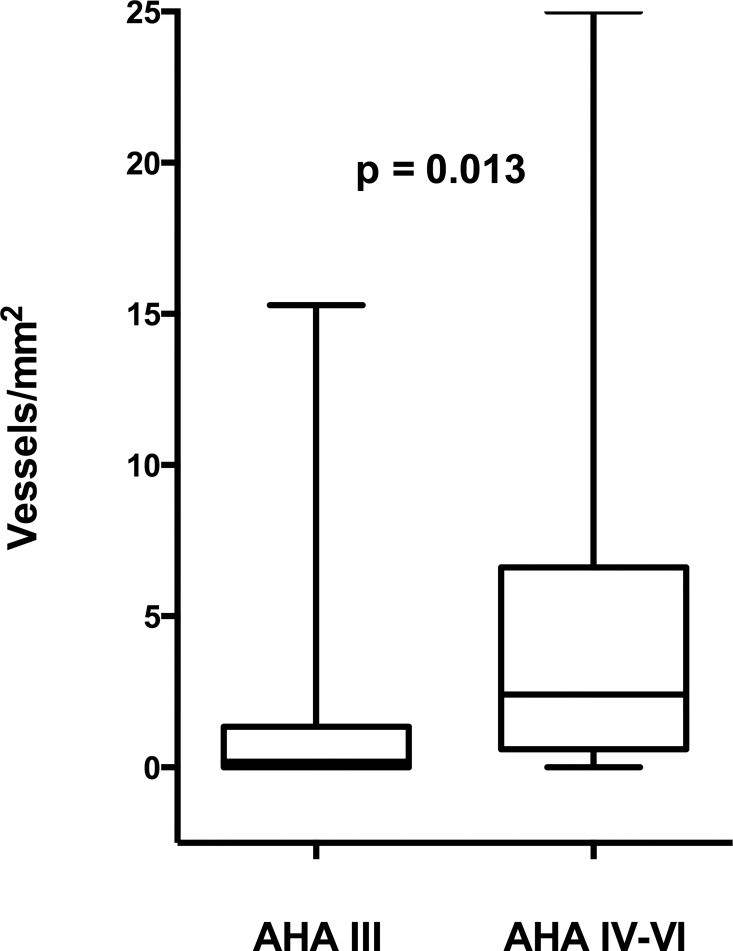
Boxplot of the number of CD34-stained vessels/mm^2^ in preatheromas (AHA III) and advanced atheromas (AHA IV-VI).

Since lesion severity differed between upstream and downstream regions of the plaque we also determined if there were differences in protein expression between plaques with different lesion severity. In the downstream region the vessel density were higher in the advanced lesion types (AHA IV to VI) compared with preatheromas (AHA III), examined as median 2.4 vessels/mm^2^ (0.6–6.5) vs. 0.2 vessels/mm^2^ (0.0–1.3), p = 0.013 (Fig 5). CD68 staining was also more intense in advanced lesions 4.2% (1.0–14.0) vs. 0.3%(0.1–1.3), p = 0.001). On the contrary the marker of hypoxia, HIF1-α, was higher in preatheromas compared to advanced lesions 6.2% (4.8–9.6) vs. 9.1% (7.1–13.2), p = 0.043. The expression of VEGF, VEGFR2 and Notch-1 were also increased in the more advanced lesions while no significant differences were found in Dll4 expression (data not shown). No correlation was found between vessel density and HIF1-α immunostaining in sections with AHA-class III (data not shown). This analysis could not be performed in the upstream region due to a low number of sections in AHA class III (n = 2).

## Discussion

Here, we show that vascularization measured *in vivo* in early atherosclerotic lesions as well as in *ex vivo* measurements from symptomatic patients with advanced atherosclerosis is increased in the upstream compared to the downstream region of the plaque. Furthermore, inflammatory markers were increased in the upstream region and we found an association between vessel density and inflammatory markers in both the upstream and downstream region. On the contrary, HIF1-α was increased in the downstream region and no association was seen with vessel density.

Intra-plaque micro vessels might provide an entry point for inflammatory cells and also cause intra-plaque hemorrhage, which might aggravate inflammation[8, 9]. Furthermore, inflammation might in itself induce vascularization[8]. Thus, a positive feedback loop between vascularization and inflammation might be present in the plaque, resulting in a vulnerable plaque phenotype.

Contrast agent uptake in carotid plaques measured with ultrasound correlates with histologically assessed plaque vascularization [21–23]. We used a newly developed and standardized, semi-automated method to quantify contrast agent uptake[15]. In this in vivo measurement, we found that contrast agent uptake was increased in the upstream regions in patients with early atherosclerotic lesions. This finding suggests that the distribution of vessel density toward upstream regions is not just a feature exclusive for more advanced, late stage plaques. Ruptures in atherosclerotic plaques are most commonly found in the upstream region[4–7] and previous cross-sectional studies reported increased contrast agent uptake in carotid plaques from patients with previous ischemic stroke[15, 22, 24, 25]. In the current study, the distribution of contrast agent uptake between upstream and downstream parts of the plaque in patients with previous ipsilateral symptoms compared with asymptomatic patients was similar. However, the low number of observations results in a low power for these analyses. Interestingly, in plaques from subjects on statin treatment, the vascular gradient was not found. This finding needs to be confirmed but may be one explanation to the plaque stabilizing action of statins.

Vascularization was increased in the upstream regions in our study as well as in sections with intra-plaque hemorrhage. This finding are in line with previous reports of increased vascularization in the shoulder regions of both asymptomatic and symptomatic carotid plaques[5, 7] and further strengthens the causal association between vascularization and IPH. Here, we confirm earlier reports when analyzing whole transverse sections of the plaque and at well-characterized distances from the maximum stenosis.

In accordance with previous studies[3, 5, 6], we found that CD68-positive macrophages were increased in the upstream region and correlated with the number of vessels. In contrast, HIF1-α-staining was increased in the downstream regions of atherosclerotic plaques. Furthermore, we found no correlation between HIF1-α and vessel density in any section. Previous studies have showed none, or even an inverse, association between HIF-α and plaque vascularization in coronary, carotid and femoral specimens[26, 27]. However, in a study by Sluimer et al., HIF1-α correlated with hypoxia as well as vessel density, VEGF and CD68 in symptomatic carotid specimens[28]. We also found that opposite to the distribution of other proteins in our study, HIF1-α immunoreactivity was increased in preatheromas (AHA III) compared with more advanced lesion types (AHA IV to VI). This explains why the total HIF1-α immunorectivity was increased in downstream regions, were AHA III lesions were predominant. Our data could be interpreted in favor of a role for HIF1-α in early stages of lesion development where increased diffusion distances leads to hypoxia, which later on stimulates vascularization. In advanced lesions however, inflammation may be the key driver of angiogenesis and vascularization is in balance with oxygen supply to the tissue.

In order to shed further light on the molecular mechanism that may be involved in plaque angiogenesis we estimated the expression of different key signaling pathways in upstream and downstream sections. VEGF and VEGFR2 are expressed in smooth muscle cells, macrophages and endothelial cells in human atherosclerosis[29]. We saw higher expression of both VEGF and VEGF-R2 in upstream sections coinciding with increased vascularization. Both VEGF and VEGF-R2 expression mainly co-localized with CD68-stained macrophages with rather low expression in endothelial cells.

We also measured Dll4 and NOTCH-1 expression since this system is a known crucial modulator of the angiogenic capacity of VEGF/VEGF-R2[12]. Both Dll4 and Notch-1 showed increased expression in upstream sections coinciding with increased vascularization. The expression of Dll4 and Notch was particularly high in areas with CD68-positive macrophages, while a weak to modest expression was found in endothelial cells. It has been proposed that Dll4 and Notch-1 are key regulators of macrophage activation in atherosclerosis[30]. Inhibition of Dll4 in LDL-receptor -/- mice led to decreased macrophage accumulation, less pro-inflammatory molecules and lessened the severity of the atherosclerotic lesions[30]. The increased expression of Dll4 and NOTCH-1 in upstream sections could thus be involved in both neo-angiogenesis as well as the inflammatory response.

## Limitations

There are some limitations to this study. The cross-sectional design of the study prevents us from drawing conclusions on casual relationships. The presence of thrombosis in some plaques may have confounded the determination of the point of maximum stenosis by MRA. As we have reported previously[6], there was however a good agreement between the narrowest lumen measured by MRA compared with histopathological examination. Since we only included CEA-patients who had undergone an MRA investigation, our population might not be representative of the population with carotid atherosclerosis in general. However, our main finding was confirmed in the CEUS examination. Due to the very fast clinical work up of patients with symptomatic carotid artery disease at our hospital we could not perform in vivo measurements of vascularization on advanced plaques.

## Conclusion

In summary, we have shown that vascularization is increased in the more vulnerable upstream regions both in earlier phases of atherosclerosis as well as in advanced symptomatic plaques. In advanced plaques, vascularization correlates with markers of inflammation but not markers of hypoxia, which we interpret in favor of inflammation being the main driver of vascularization in these plaques while hypoxia might be more important in early lesions. The association with IPH may suggest a causative relation to plaque vulnerability.
